# Comparison of ego strength between aggressive and non-aggressive alcoholics: a cross-sectional study

**DOI:** 10.3325/cmj.2018.59.156

**Published:** 2018-08

**Authors:** Zrnka Kovačić Petrović, Tina Peraica, Dragica Kozarić-Kovačić

**Affiliations:** 1 University of Zagreb School of Medicine, Zagreb, Croatia; 2 University Psychiatric Hospital Vrapče, Zagreb, Croatia; 3 Department of Psychiatry, University Hospital Dubrava, Referral Center for Stress-related Disorders of the Ministry of Health of the Republic of Croatia, Zagreb, Croatia; 4 University Department of Forensic Sciences, University of Split, Split, Croatia

## Abstract

**Aim:**

To determine the differences between aggressive and non-aggressive alcoholics in sociodemographic and clinical characteristics and ego strength.

**Methods:**

This cross-sectional study included 111 aggressive and 123 non-aggressive male alcoholics aged between 25 and 60 years who were admitted to the Department for Alcoholism, University Psychiatric Hospital Vrapče, Zagreb, Croatia, from January to December 2016. All participants met the diagnostic criteria for alcohol dependence according to the Croatian Mini International Neuropsychiatric Interview (MINI), 4th revised edition of Diagnostic and Statistical Manual of Mental Disorders, and the International Classification of Diseases. Participants were clinically assessed by an experienced psychiatrist using a clinical interview, MINI, Questionnaire from the Brown-Goodwin Lifetime History of Aggression, and Ego Identity Scale (EIS) according to Erikson. A clinical psychologist performed cognitive function measurements. EIS scores were analyzed using one-way analysis of variance.

**Results:**

In comparison with non-aggressive alcoholics, aggressive alcoholics were more often divorced, unemployed, hospitalized, and first treated for alcoholism at an earlier age (*P* < 0.05 for all). They more frequently experienced depression (42.4% vs 19.4%, *P* = 0.013) and attempted suicide (34.7% vs 6.2%, *P* = 0.003), achieved a lower level of maturity at the second stage of psychosocial development related to shame and doubt (14.0 ± 4.1 vs 17.4 ± 3.7, *P* = 0.013) and at the fourth stage related to inferiority (13.1 ± 6.8 vs 18.1 ± 9.3, *P* = 0.011), and had lower total EIS score (75.8 ± 20.4 vs 85.2 ± 21.5, *P* < 0.012) than non-aggressive alcoholics.

**Conclusion:**

Aggressive alcoholics had weaker ego-strength than non-aggressive alcoholics, experienced more depressive reactions and suicide attempts, and showed poorer psychosocial functioning.

Oxford Centre for Evidence-based Medicine level of evidence: 3**

Alcohol-related aggression is often present in the context of chronic alcohol consumption and dependence. Up to 50% of alcohol-dependent men display violent behavior (1), and alcohol dependence and abuse are the second most commonly diagnosed cause of suicide (after depression) (2). Alcohol-related aggression is an ambiguous phenomenon, influenced by individual characteristics, such as male sex, personality, high irritability, and lack of empathy, as well as by the interaction of social and neurobiological factors (2).

Recent literature describes alcoholics according to their personality characteristics, type of drinking, psychopathology, and psychological characteristics (3-5). The main psychodynamic characteristics of alcoholics are neuroticism, weak ego, addiction, and personality changes (6).

In alcoholics, there is ample evidence on weak ego, psychopathological traits, antisocial behavior, hostility as a sign of poor control of drives, impulsivity, low frustration tolerance, difficulties in establishing adequate relationships, problems with sexual identity, and negative self-image (7-19).

Successful treatment of an alcoholic depends on the improvement of his or her ego strength (20). Ego strength is defined as the capacity for positive attitude toward oneself and one’s abilities, self-esteem, emotional flexibility, relationships, and social interactions (21). Lower ego strength in aggressive alcoholics is an indication of poor compliance, and the capacity of the ego to neutralize aggression is a measure of the ego strength and maturity (22,23). Erikson’s theory (24,25) describes the stages of ego development throughout lifetime and describes alcoholics as individuals with a negative ego identity that decreases and destroys their abilities.

Ego strength was already investigated in aggressive alcoholics, indicating that alcoholics with weak ego cannot overcome the problem correctly (26-28). Therapeutic approach to this group of alcoholics is quite demanding, but limited existing results are inconsistent. We tested the hypothesis that aggressive male alcoholics have weaker ego’s capacity, and different socio-demographic and clinical characteristics from non-aggressive male alcoholics. Thus, the aim of this study was to determine the differences between aggressive and non-aggressive male alcoholics in their sociodemographic and clinical characteristics and ego strength at different stages of psychosocial development.

## Patients and methods

### Patients

**Figure 1:**
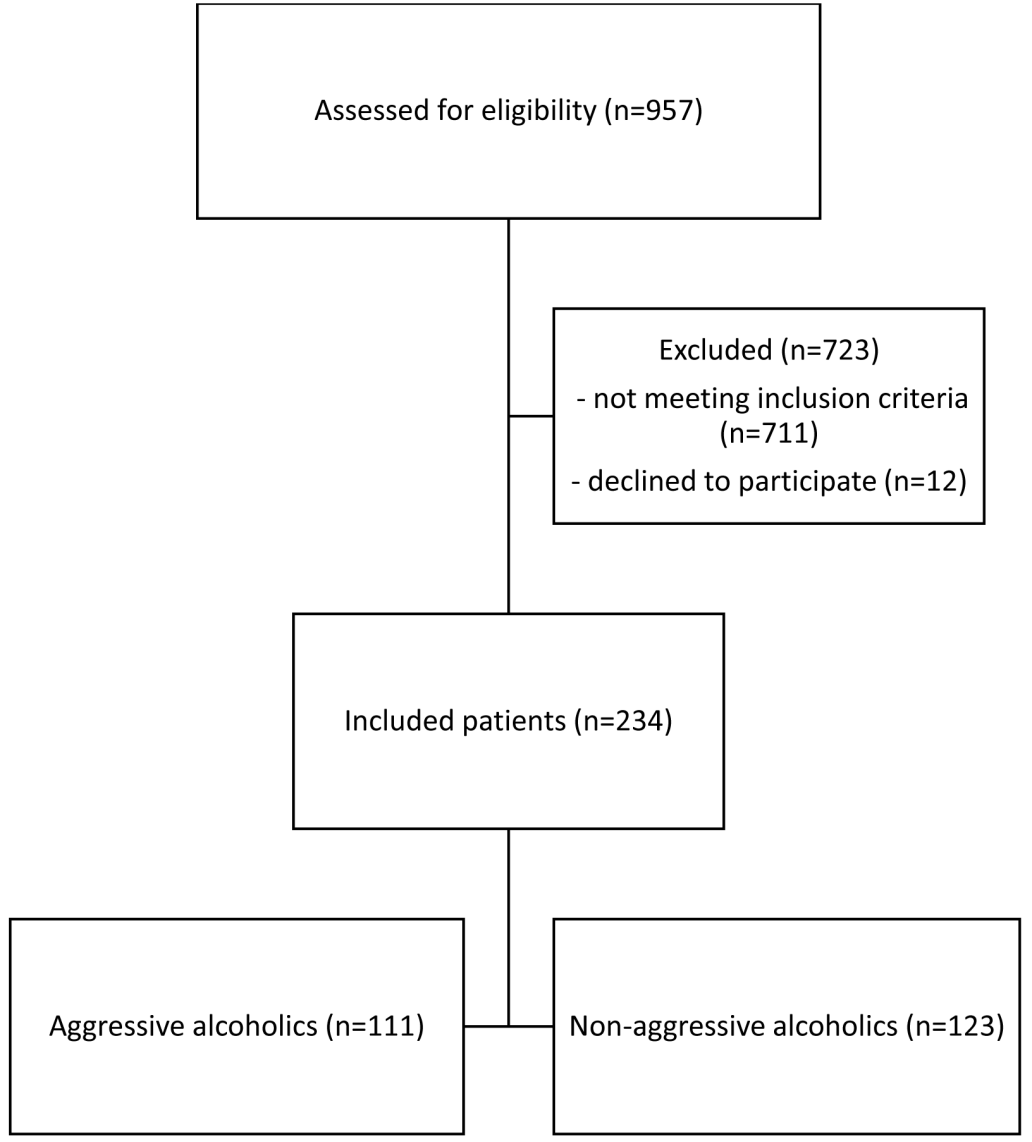
Study flow diagram.

This single-center, cross-sectional study was conducted in 111 aggressive and 123 non-aggressive male alcoholics aged between 25 and 60 years, who were selected out of a total of 957 male alcoholics admitted to the Department for Alcoholism, University Psychiatric Hospital Vrapče, Zagreb, Croatia from January 2016 to December 2016 (Figure 1). All patients had been drinking regularly for at least 5 years. Of 723 excluded patients, 12 declined to participate in the study and 711 were excluded because of multiple somatic or psychiatric comorbidity. Alcoholics with a somatic illness (n = 594), abuse of drugs or other psychoactive agents in the previous year (n = 125), organic difficulties (n = 185), schizophrenia or affective disorder of non-alcoholic etiology (n = 45), antisocial disorder before the onset of alcoholism (n = 76), and other primary mental disabilities affecting intelligence (n = 61) were excluded (Figure 1).

Upon admission, participants were given psychopharmacological therapy for 8 days to remove alcohol withdrawal symptoms. The participants were evaluated between Day 10 and Day 15 of hospitalization.

### Method

The Croatian version 5.0.0 of the Mini International Neuropsychiatric Interview (MINI) (29) was used to evaluate psychiatric morbidity in all participants who met the diagnostic criteria for alcohol dependence according to the Diagnostic and Statistical Manual of Mental Disorders, 4th edition – text revision (DSM-IV-TR) (30) and the International Classification of Diseases ICD-X (31). Clinical assessment and data collection was performed by an experienced psychiatrist using MINI (29), structured clinical interviews (for collection of demographic data and data related to alcoholism), Questionnaire on History of Antisocial Behavior adapted from Brown-Goodwin Lifetime History of Aggression (32), and Ego Identity Scale according to Erikson (33). A clinical psychologist performed cognitive function measurements.

The study was approved by the Ethics Committee of the Department of Psychiatry, University Psychiatric Hospital Vrapče (May 29, 2015; approval No. 23-35/4-15 Ei 12/15). All participants gave their written informed consent to participation in the study and presentation of the data in the manuscript.

The study was designed to ensure the correct treatment and safety of the participants in accordance with Good Clinical Practice (34), Declaration of Helsinki (35), Health Care Act (36), and Law on Protection of Patients’ Rights (37). Before providing their written informed consent, the participants were explained the purpose of the study and informed that their participation was completely voluntary, that they would receive no financial or any other type of remuneration for participation, and that they were free to withdraw from the study at any time without the need to specify the reason and without any consequences for their further treatment. For all additional information, they could talk to the study investigators. The identity of each participant was kept confidential and data were protected.

### Instruments

The following instruments were used for assessment of each participant:

Structured clinical interview for demographic data collection was used to collect data on age, education level, employment status, marital status, and number of children.

Structured clinical interview for history of alcoholism consisted of questions on the age of drinking onset, age of regular and excessive drinking onset, age of first treatment received for alcoholism, and age when the first alcoholism-related problems occurred. Regular and excessive drinking was defined as daily consumption of at least 70 g of ethanol for at least six months. Further questions were related to the type of alcoholic beverages consumed in the last four weeks, type of alcoholic beverages consumed before and after the age of 20, duration of alcohol abuse, number of hospitalizations related to alcohol abuse, occurrence of depression, and suicide attempts. Occurrence of depression related to drinking was taken into consideration only if the participant was hospitalized or treated for depression with antidepressants. There were also questions related to alcoholism in participants’ parents. As alcoholics often rationalize, deny, and negate the use of alcohol, data collected from the participants were compared with the data provided by their family members for confirmation and such data were included and kept in the participant’s medical records.

Questionnaire from the Brown-Goodwin Lifetime History of Aggression (32) was used to distinguish alcoholics with aggressive behavior from those without such behavior. The purpose was to collect information on behavior problems, such as discipline problems in the army, discipline problems in the workplace, attacking other people, destroying property, arrests for violent behavior, arrests for other crimes, and committed crimes that did not result in an arrest. In each category, no event was scored 0, one event was scored 1, two events were scored 2, three or frequently occurring events were scored 3, four or more events were scored 4. Aggressive behavior before the age of 15 was not included in scoring to avoid scoring aggression during specific developmental phases. Furthermore, the diagnosis of personality disorder should be finally established after the age of 18. Participants who scored 8 or more were considered to have the life history of aggressive behavior. This questionnaire was filled out by the investigator rather than the participants, for higher data reliability. The collected data on aggressive behavior were compared with the data obtained from the family for confirmation.

Wechsler Adult Intelligence Scale test was applied to assess cognitive functions and exclude participants with more severe psycho-organic disorders (internal consistency varied from 0.90 to 0.97 and test-retest reliability was *r* = 0.75) (38).

Ego Identity Scale according to Erikson (33) was used to assess the level of fixation in psychosocial development. The scale consists of 80 statements. Eight statements are used for the assessment of the level of honesty (scale of lies), and 72 statements in groups of 12 are related to six of the eight developmental stages, starting from early childhood to the adulthood. After receiving instructions, the participants filled out this questionnaire themselves. Ego Identity Scale according to Erickson was validated in the Croatian population and showed high validity (internal consistency, alpha = 0.73 and test-retest reliability, *r* = 0.84) (16,26).

### Statistical analysis

According to literature (39), a small to moderate effect size of 0.4 was to be expected in this field of research. A priori power analysis in G*Power program (*http://www.gpower.hhu.de/en.html*) was performed using the following parameters: *t* test for independent samples, alfa error = 0.05 and 1-beta error = 0.8. Using this effect size in the sample size calculation for two independent samples, we needed 100 patients per study group. To compensate for the expected high drop-out rate due to exclusion criteria, eligible patients (N = 957) were oversampled to ensure the adequate net sample size of 111 and 123 in the aggressive and non-aggressive alcoholic groups, respectively.

Data were normally distributed for ego variables and age, as shown by Kolmogorov-Smirnov test and presented as percentages or means and standard deviations (SD). Differences in numerical variables (duration of alcohol consumption, duration of alcohol dependence, age of drinking onset) between groups, which were not normally distributed, were analyzed with non-parametric tests. Differences between groups were tested with Kruskal-Wallis one-way analysis of variance by ranks. Every test was followed by multiple comparison of mean ranks between groups (Mann-Whitney U test) and the resulting *P* values were corrected by Bonferroni method. Categorical (descriptive) variables were compared between groups with non-parametric Pearson χ^2^-test, while differences between numerical (quantitatitve) variables were tested with *t-*test with Bonferroni correction. One-way analysis of variance (ANOVA) with repeated measures was used to test for the differences in ego strength variables. Bonferroni correction was used to assess type 1 error. The level of statistical significance was set at 0.01 and the statistical analysis was performed using a free statistics program JASP, version 0.8.6 (2018, JASP Team, Amsterdam, the Netherlands).

## Results

There were no significant differences in age (*P* = 0.442) and education level (*P* = 0.152) between aggressive and non-aggressive alcoholics (Table 1). Majority of participants (43.8%) completed high-school education, 27.7% completed elementary education, 16.1% did not complete elementary education, 7.1% completed college, and 5.4% had a university degree. A larger number of non-aggressive alcoholics were married or cohabiting and fewer of them were divorced (Table 1). Aggressive alcoholics were more often unemployed and welfare beneficiaries, whereas non-aggressive alcoholics were more often farmers. They also differed in the number of children (Table 1).

**Table 1:** Demographic and clinical characteristics of aggressive and non-aggressive alcoholics

Characteristics*	Alcoholics		*P*
	aggressive (n = 111)	non-aggressive (n = 123)	
Age (years; mean ± standard deviation [SD])	45.4 ± 8.2	47.9 ± 8.1	0.442†
Education (years; mean±SD)	12.3 ± 2.1	11.7 ± 1.4	0.152†
Duration of alcohol consumption (years; median [Q1-Q2])	25 [20-28]	24 [21-27]	0.123‡
Duration of alcohol dependence (years; median [Q1-Q2])	14 [11-20]	13 [10-19]	0.419‡
Age of drinking onset (years; median [Q1-Q2])	15 [10-19]	16 [12-21]	0.118‡
Age of first alcohol-related treatment (years; mean±SD)	28.4 ± 7.5	36.0 ± 7.1	0.011†
Marital status (%)			0.012^§^
single	12.0	25.8	
married or cohabiting	42.0	62.9	
divorced	36.0	9.7	
widowed	10.0	1.6	
Employment status (%)			0.019^§^
employed	66.1	74.2	
unemployed	19.9	8.1	
retired (age-related)	0	1.6	
retired (disability-related)	3.9	4.8	
welfare recipient	9.1	0	
farmer	1.0	11.3	
Number of children (%)			0.017^§^
none	21.9	33.9	
one	34.1	12.9	
two	34.1	45.2	
three	7.9	6.5	
four or more	2.0	1.6	
Usual type of alcoholic beverage before the age of 20 (%)			0.003^§^
distilled	28.4	2.9	
wine	7.8	8.6	
Noah wine	7.8	28.6	
beer	44.1	22.9	
mixture of the above	11.9	37.1	
Usual type of alcoholic beverage after the age of 20 (%)			0.586^§^
distilled	22.4	14.5	
wine	11.6	6.5	
Noah wine	9.8	6.5	
beer	8.2	11.3	
mixture of the above	48.0	61.3	
Parental alcoholism (%)			0.488^§^
father	54.5	38.7	
mother	2.1	4.8	
both	11.9	12.9	
neither	31.5	43.5	
Number of hospitalizations (%)			0.012^§^
1-5	19.8	67.8	
6-10	56.2	19.1	
11-15	14.7	7.9	
16-20	7.3	4.8	
21-25	1.5	0.4	
26-30	0.5	0	
Suicide attempt (%)	34.7	6.2	0.003^§^
Depression (%)	42.4	19.4	0.013^§^

*Percentages are based on the number of patients with data.

†*t*-test.

‡U test.

§χ2-test.

There was no difference in the duration of alcohol consumption or dependence, age of drinking onset, type of alcoholic drinks usually consumed after the age of 20, and parental alcoholism (Table 1). However, in comparison with non-aggressive alcoholics, aggressive alcoholics received their first alcoholism treatment at a significantly earlier age and more often drank distilled alcoholic beverages and beer, whereas non-aggressive alcoholics consumed wine (especially from the Noah grape) and mixed beverages. Rates of hospitalization, depression, and suicide attempts were significantly higher in aggressive than non-aggressive alcoholics.

Differences in psychosocial health according to the Ego Identity Scale scores between aggressive and non-aggressive alcoholics were tested using ANOVA (Table 2). In comparison with non-aggressive alcoholics, aggressive alcoholics achieved significantly lower psychosocial maturity in the second (EIS2) and fourth (EIS4) stages and had lower total scores. No significant differences were found between the groups in other stages of psychosocial maturity, although aggressive alcoholics scored on average higher than non-aggressive alcoholics.

**Table 2:** Differences in psychosocial health according to the Ego Identity Scale (EIS)*

	Score (mean±SD)			
Variables	aggressive alcoholics (n = 111)	non-aggressive alcoholics (n = 123)	ANOVA (F) d.f. = 2.368; power = 0.057	*P*
EISL	7.1 ± 3.8	6.5 ± 3.7	96.19	0.581
EIS1	10.3 ± 3.7	10.5 ± 5.8	212.34	0.723
EIS2	14.0 ± 4.1	17.4 ± 3.7	156.37	0.013
EIS3	12.8 ± 4.1	12.7 ± 1.1	235.31	0.273
EIS4	13.1 ± 6.8	18.1 ± 9.3	147.15	0.011
EIS5	13.1 ± 8.4	12.5 ± 9.1	263.14	0.327
EIS6	13.7 ± 9.5	14.0 ± 8.3	254.25	0.524
EISTOT	75.8 ± 20.4	85.2 ± 21.5	145.31	0.012

*SD – standard deviation, ANOVA – analysis of variance, EISL – Lie scale, EIS1 – first crisis phase (basic trust vs mistrust), EIS2 – second crisis phase (autonomy vs shame, doubt, and insecurity), EIS3 – third crisis phase (initiative vs guilt), EIS4 – fourth crisis phase (industry vs inferiority), EIS5 – fifth crisis phase (identity vs role diffusion), EIS6 – sixth crisis phase (intimacy vs isolation), EISTOT – total score.

## Discussion

The main findings of our study are that aggressive alcoholics were more often divorced, unemployed, hospitalized, and first treated for alcoholism at an earlier age than non-aggressive alcoholics. They also experienced depression and attempted suicide more often and had weaker ego strength than non-aggressive alcoholics. These findings are in line with our hypothesis and consistent with the limited existing literature on psychological characteristics of aggressive alcoholics (40).

According to both our study and other research, aggressive alcoholics are the most often hospitalized alcoholics and most commonly referred to as “alcohol frequent attenders,” or “high-impact users” and “high-volume users” (41-43). A high percentage of our participants experienced depression and attempted suicide, which also corresponds to previous findings (44). Although persons with alcohol use disorders often have co-occurring psychiatric disorders, they rarely receive specialized substance abuse treatment that addresses both conditions. Aggression, impulsivity, alcoholism severity, hopelessness, and negative affect in alcoholics are predisposing factors for suicide, which often takes place in the context of a depressive episode (45).

Hostility (aggressiveness), often present in alcoholics, is a sign of poor impulse control and weak ego (4,6,8,14,18,20,23,46). However, findings related to the differences between aggressive and non-aggressive alcoholics in ego strength across developmental stages are inconsistent (16,26-28). The success of the treatment of an alcoholic depends on the increase in their ego strength (46). Still, increase in ego strength is difficult to achieve in persons with alcohol-related aggression due to high levels of impulsiveness (47,48) and low stress tolerance (49,50). Persons with impaired inhibitory control of their behavior, who cannot delay gratification or tolerate unpleasant emotions, often become aggressive when intoxicated. Their treatment should focus on increasing their ego strength by improving cognitive and emotional control.

The use of Ego Identity Scale according to Erickson to measure ego strength allowed us to establish the developmental stage at which the disorder occurred and determine whether or not a certain personality trait was completely developed. We found that aggressive alcoholics, in comparison with non-aggressive alcoholics, achieved lower level of psychosocial maturity in the second (EIS2) and fourth (EIS4) stages and had lower total scores. EIS2 measures the second stage of psychosocial maturity according to Erikson and relates to autonomy and shame, whereas EIS4 measures the fourth stage and relates to industry or inferiority. Kozarić-Kovačić (16,26) also found lower levels of maturity in the fourth stage of psychosocial development in alcoholics involuntarily admitted to hospital for aggressive behavior in comparison with non-aggressive alcoholics and in alcoholics who committed violent crimes in comparison with those who did not. Žarković-Palijan et al (28) obtained similar results for alcoholics who committed homicide in comparison with those who committed felony traffic offenses and burglaries. According to Erikson’s psychosocial theory, the fourth stage occurs when the main basis for a definitive character formation develops, representing “a leap from internal rebellion to new knowledge” (24,25). This is the period in which the identity confusion, which stems from the “inability or lack of opportunity to learn,” could be prevented. According to psychodynamic theories, this stage ends with successful or unsuccessful sublimation and development of a total object relation (22). Nenadić-Šviglin (27) found no significant differences between aggressive and non-aggressive alcoholics in any of the stages of psychosocial development, which may be explained by a relatively small sample and participants selection method.

The Erickson's fourth stage of psychosocial development is critical for the development of self-confidence in children as they need to cope with new social and school demands. Success leads to a sense of competence, while failure results in feelings of inferiority.

In our study, aggressive alcoholics had lower ego strength in the second stage of psychosocial development, which had not been reported in earlier studies (16,26-28). In this stage, a child develops a sense of personal control over physical skills and a sense of independence. Success leads to feelings of autonomy, whereas failure results in feelings of shame and doubt. This stage is very important in the formation of shame, doubt, and insecurity as a reaction to developmental damage; otherwise, autonomy develops (24,25). Autonomy concerns children’s sense of mastery over themselves and over their drives and impulses.

Significant differences found between aggressive and non-aggressive alcoholics in the second and fourth stage of psychosocial development in our study could be related to the developmental disturbance of the ego in this period, with implications on the total ego strength. Since the second psychosocial developmental stage is important for the formation of autonomy and identity, and the fourth stage is associated with competence and inferiority in ego formation, the question is to what extent these periods contribute to the potentially weaker ego in aggressive alcoholics, their lower control of aggressive-hostile impulses, and tendency to use aggressive defense and behavior, which is even more emphasized in intoxicated state due to secondary ego-inhibition caused by alcohol.

Although it is well known that alcoholism is a shame-based syndrome, publications on shame are largely absent from the literature on alcoholism. There is no research specifically related to the developmental psychological understanding of this problem, because most studies are based on cognitive, behavioral, or biological approaches. According to Tomkins’ theory, there is an association between shame and anger and laughter (51-53). Tomkins noted that learned anger (the combination of innate anger and learned display of anger) is used to alter the interpersonal field. Thus, aggression may be one of the expressions of anxiety in situations where one feels ashamed and protects oneself against excessive shame by rage and aggression (54).

Our study has several limitations. It was a single-center study and it included only male alcoholics, because there are generally fewer women alcoholics, especially aggressive ones, who are admitted for the treatment of alcoholism. Furthermore, the design of the study was cross-sectional, which precludes causal inferences. However, the sample size was large enough to detect the differences, data were collected by a psychiatrist and psychologist with experience in the field of alcohology, and the instruments used were valid and standardized questionnaires.

In conclusion, the therapeutic approach should take into account the characteristics and weaker ego of aggressive alcoholics and be directed at ego strengthening during their alcoholism treatment (55,56). Our results implied more pronounced shame in aggressive alcoholics, who mostly use aggressive behavior as a defense mechanism to hide it. This may be a reason why aggressive alcoholics are so demanding and needy as psychotherapeutic patients, often provoking extremely strong reactions in their therapists.
